# A Comparative Study of Electrolyte Types and Processing Parameters in the Electrochemical Oxidation of Carbon Fibers and Their Interfacial Interactions with Vinyl Ester Resin

**DOI:** 10.3390/ma19204382

**Published:** 2026-10-19

**Authors:** Chuting Zhang, Ruirui Yang, Wantao Guo, Jinhao Zhang, Pengjun Cao, Wanshuang Liu

**Affiliations:** 1 Shanghai High Performance Fibers and Composites Center (Province-Ministry Joint), Shanghai Key Laboratory of Lightweight Composite, College of Textiles, Donghua University, 2999 North Renmin Road, Shanghai 201620, China; zzctzct@163.com (C.Z.); 19036813620@163.com (J.Z.); 2 Luoyang Ship Material Research Institute, 169 South Binhe Road, Luoyang 471023, Chinac_pengjun@163.com (P.C.)

**Keywords:** carbon fibers, vinyl ester resin, electrochemical oxidation, processing parameters, interfacial interactions

## Abstract

The chemically inert surface of carbon fibers (CFs) limits their interfacial adhesion with vinyl ester resin (VER). In this study, electrochemical surface treatment was performed in four electrolyte systems with different acid–base characteristics, including H_2_SO_4_, NH_4_HSO_4_, NH_4_HCO_3_, and KOH. The effects of electrolyte type, concentration, current density, and treatment time on CF surface properties and CF/VER interfacial performance were evaluated. Acidic electrolytes produced stronger oxidative functionalization, increasing the surface oxygen content from 13 at.% to 27 at.% and 26 at.% for H_2_SO_4_- and NH_4_HSO_4_-treated CFs, respectively. All treatments significantly improved wettability, reducing the water contact angle from 116.1° to 30.2–49.4° and the VER contact angle from 121.9° to 29.4–50.4°. Consequently, the interfacial shear strength increased from 27.9 MPa to 42.8–48.4 MPa, while the single-fiber tensile strength remained at 3.1–3.3 GPa. The enhancement was attributed to the synergistic effects of chemical oxidation and mild physical etching. These results clarify the distinct roles of acidic and alkaline electrolytes and provide guidance for optimizing electrochemical surface treatment of CFs for high-performance CF/VER composites.

## 1. Introduction

Carbon fiber-reinforced thermosetting polymer composites (CFRTPs) offer high specific strength and specific modulus, low density, excellent fatigue resistance, good dimensional stability, and strong resistance to corrosion and chemical degradation [1,2]. Among CFRTPs, carbon fiber-reinforced vinyl ester composites (CFRVEs) are particularly suitable for marine structures, chemical storage tanks and pipelines, wind turbine components, automotive parts, and other lightweight structures exposed to corrosive or humid environments [3,4,5,6], owing to the excellent chemical resistance, moisture resistance, toughness, and relatively low processing cost of vinyl ester resins (VERs) [7]. The interface between carbon fibers (CFs) and the resin matrix plays a critical role in stress transfer and strongly influences the mechanical performance, damage tolerance, and durability of CFRTPs. Strong interfacial adhesion enables efficient load transfer, whereas a weak interface can lead to fiber–matrix debonding and premature composite failure. To date, studies on the interface behavior of CFRTPs have predominantly focused on CFs and epoxy matrix, whereas limited attention has been devoted to the interface between CFs and VERs [8,9,10]. Pristine CFs possess highly ordered graphitic structures and typically exhibit chemically inert surfaces with low polarity and low surface free energy [11,12,13], leading to the poor interfacial compatibility and limited interfacial adhesion with the VER matrix. Such interfacial deficiencies would deteriorate the overall mechanical performance of CFRVEs and pose a critical challenge for their further performance enhancement and engineering applications [14].

To address the weak interfacial interactions between CFs and resin matrices, various surface modification techniques have been developed, including plasma treatment [15,16], surface coating [17], chemical grafting [18], and electrochemical oxidation [19,20,21]. Among these approaches, plasma treatment can introduce polar functional groups and increase the surface energy of CFs under relatively mild conditions. However, the stability and uniformity of the modified surface depend strongly on the treatment conditions. Surface coating, including sizing agents, coupling agents, and inorganic coatings, can provide a tailored interphase between CFs and the resin matrix. Such coatings can enhance fiber–matrix interactions through improved wetting, chemical bonding, and mechanical interlocking. Chemical grafting is another effective strategy for modifying CF surfaces through the covalent attachment of functional groups or polymer chains, thereby promoting stronger fiber–matrix interactions through chemical bonding and improving interfacial compatibility. However, this approach generally requires relatively complex reaction procedures. In contrast, electrochemical oxidation combines surface functionalization with controllable topographical modification and is particularly attractive for continuous industrial processing because of its simple operation and tunable treatment intensity [22,23]. In this process, CFs serve as the anode under an applied electric field, and anodic oxidation occurs in the electrolyte [24]. Consequently, oxygen-containing polar functional groups, such as hydroxyl, carbonyl, and carboxyl groups, are introduced onto the fiber surface, accompanied by moderate surface etching and topographical regulation [25]. These chemical and morphological changes increase the surface free energy and wettability of the CFs, thereby enhancing their interfacial adhesion with the resin matrix [26,27].

In recent years, considerable research effort has been devoted to the electrochemical oxidation of CFs in various electrolyte systems. Previous studies have shown that acidic electrolytes, such as H_2_SO_4_, can substantially increase the surface oxygen content and promote the formation of oxygen-containing functional groups, particularly carboxyl groups [24,28]. In contrast, alkaline electrolytes, such as NH_4_HCO_3_ and KOH, promote surface activation and mild etching of CFs. Both acidic and alkaline electrolyte systems have been reported to improve the surface free energy, wettability, and interfacial performance of carbon fiber-reinforced composites, although to different extents [19,22,29]. Moreover, continuous electrochemical oxidation has been shown to regulate the surface topography and increase the concentration of active functional groups while largely preserving the intrinsic mechanical properties of CFs, thereby enhancing their interfacial interactions with resin matrices [25]. However, most previous studies have focused on individual electrolyte systems or the optimization of specific processing parameters. A systematic study of how electrolyte type and processing parameters affect the surface structure and properties of CFs remains limited. Establishing clear structure–property relationships among electrolyte characteristics, surface chemistry, wettability, and interfacial performance is essential for the precise control of carbon fiber surface modification and the development of high-performance carbon fiber-reinforced composites.

In this work, a systematic comparison of different electrolyte systems and processing parameters was conducted to elucidate their effects on the surface chemistry, morphology, wettability, and interfacial performance of CFs with the VER matrix. In particular, distinct oxidation behaviors, surface structural evolution, and interfacial enhancement mechanisms associated with different electrolyte systems were further revealed. Four representative acidic and alkaline electrolytes, including sulfuric acid (H_2_SO_4_), ammonium bisulfate (NH_4_HSO_4_), ammonium bicarbonate (NH_4_HCO_3_), and potassium hydroxide (KOH), were selected to investigate the effects of electrolyte type, electrolyte concentration, current density, and treatment time on the surface structure and properties of CFs. The surface morphology, roughness, graphitic/crystalline structure, chemical composition, surface free energy, wettability, single-fiber mechanical properties, and CF/VER interfacial bonding performance were systematically characterized to establish the relationships between electrochemical treatment parameters and the resulting surface and interfacial properties. Furthermore, the roles of electrolyte chemistry in regulating CF oxidation, surface structural evolution, and interfacial reinforcement were clarified, providing a basis for optimizing continuous electrochemical surface treatment and developing high-performance CFRVEs.

## 2. Experimental

### 2.1. Materials

Unsized CFs used in this study were supplied by Inner Mongolia Guangwei Carbon Fiber Co., Ltd., Baotou, China. Bisphenol A-based VER and a curing agent (methyl ethyl ketone peroxide, MEKP) were supplied by China Huachang Polymer Co., Ltd., Shanghai, China. Sulfuric acid (H_2_SO_4_), ammonium bisulfate (NH_4_HSO_4_), ammonium bicarbonate (NH_4_HCO_3_), and potassium hydroxide (KOH) were purchased from Shanghai Titan Scientific Co., Ltd., Shanghai, China.

### 2.2. Electrochemical Surface Treatment of CFs

**Figure 1:**
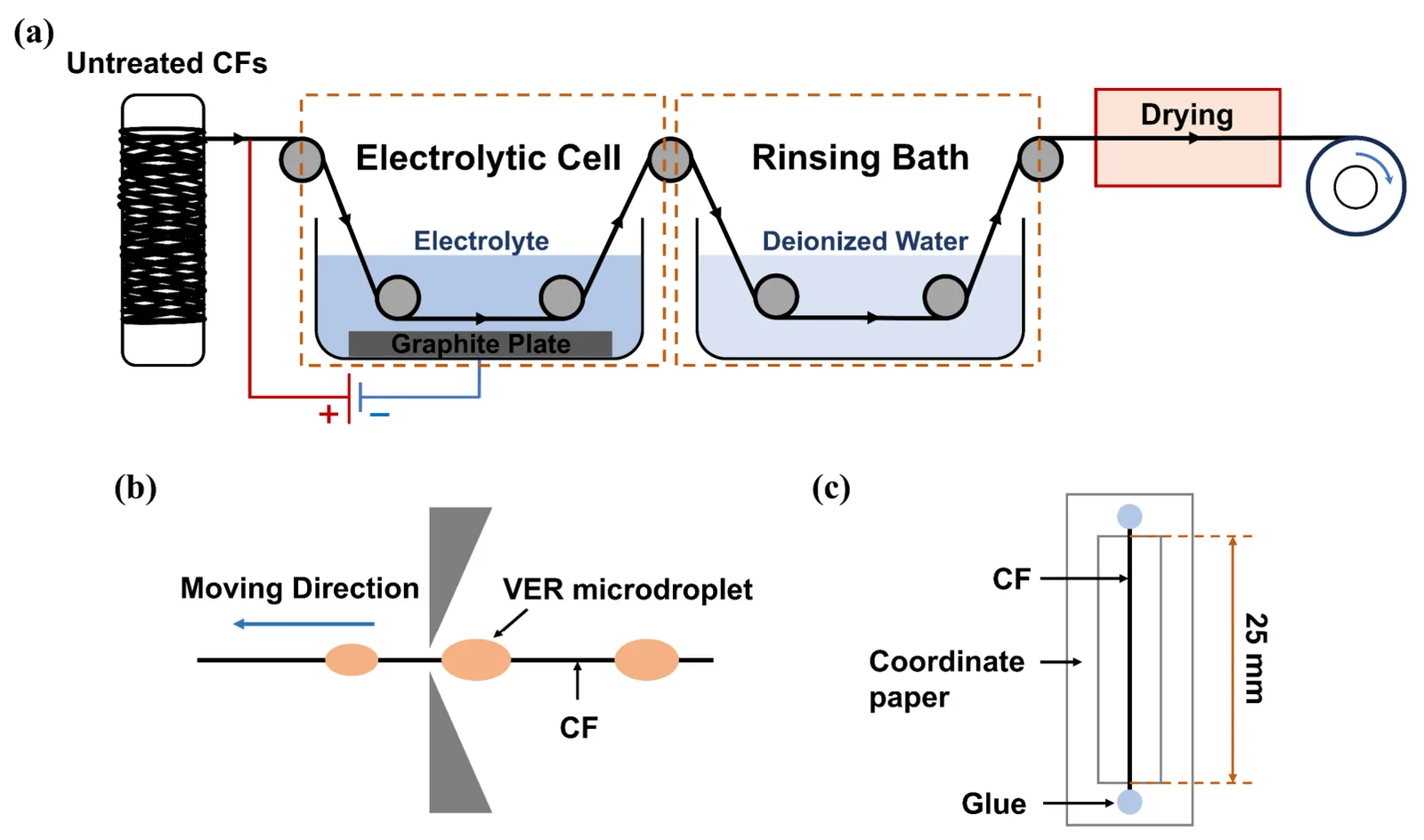
Schematic illustrations of experimental procedures and characterization methods: (**a**) electrochemical surface treatment setup for CFs, (**b**) microdroplet debonding test, and (**c**) single-fiber tensile test.

The untreated CFs were used as the anode, while a graphite plate served as the cathode. Electrochemical surface treatment was performed in various electrolyte systems with different concentrations using the experimental setup shown in Figure 1a. The electrolyte systems included sulfuric acid (H_2_SO_4_, 0.2, 0.5, and 1.0 wt%), ammonium bisulfate (NH_4_HSO_4_, 0.5, 1.5, and 3.0 wt%), ammonium bicarbonate (NH_4_HCO_3_, 1.5, 3.0, and 5.0 wt%), and potassium hydroxide (KOH, 2.0, 5.0, and 8.0 wt%). During electrochemical treatment, the applied current densities were 3, 5, and 10 A/m^2^, and the treatment times were set to 30, 60, and 120 s. The applied current was calculated based on the exposed surface area of the carbon fiber tow, which was estimated from the single-filament diameter, filament number, and immersed fiber length. First, the electrolyte concentration was optimized at a fixed current density of 5 A/m^2^ and a treatment time of 60 s. Subsequently, the current density was optimized using the selected electrolyte concentration while maintaining the treatment time at 60 s. Finally, the treatment time was optimized based on the optimized electrolyte concentration and current density. After electrochemical treatment, the CFs were thoroughly rinsed with deionized water, collected, ultrasonically cleaned in deionized water for 30 min to remove residual electrolyte and surface contaminants, and then dried in an oven at 80 °C for 60 min.

### 2.3. Characterization

The surface morphology of the CFs was characterized by scanning electron microscopy (SEM, Sigma 360, ZEISS, Oberkochen, Germany). Prior to SEM observation, the samples were sputter-coated with gold. SEM images were acquired at an accelerating voltage of 5 kV and a working distance of approximately 9 mm. The microscale surface topography and roughness of the CFs were further characterized by atomic force microscopy (AFM, Dimension FastScan, Bruker Corporation, Ettlingen, Germany). For AFM sample preparation, double-sided adhesive tapes were attached to both sides of a glass slide, and individual CF filaments were straightened and fixed on the tapes. The prepared samples were then mounted on the AFM sample stage. AFM measurements were performed in tapping mode at a scan rate of 1 Hz over an area of 1 μm × 1 μm. The surface roughness was quantified using the arithmetic mean roughness (Ra), which represents the average absolute deviation of the surface height from the mean plane, as calculated from the AFM height images.

The graphitic structure of the CFs was analyzed by Raman spectroscopy using a Raman spectrometer (LabRAM HR Evolution, HORIBA, Palaiseau, France). A bundle of CFs was fixed on a glass slide, and the spectra were collected using a 532 nm laser under a 100× objective lens in the range of 750–1950 cm^−1^. The Raman spectra were fitted to obtain the intensities of the D and G bands, and the intensity ratio of the D band to the G band (ID/IG) was used to evaluate the degree of surface defects in CFs.

The crystalline structure of the CFs was characterized by X-ray diffraction (XRD, D8 Advance, Bruker, Ettlingen, Germany). The interlayer spacing of the graphitic (002) plane (d_002_) was calculated according to Bragg’s equation:

(1) $$d_{002}=\frac{\mathrm{n\lambda}}{2\mathrm{sin\theta}}$$

where *d*_002_ is the interlayer spacing of the graphitic (002) plane, *n* is the diffraction order, *λ* is the X-ray wavelength, and *θ* is the Bragg diffraction angle.

The crystallite size along the c-axis (L_C_) was calculated using the Scherrer equation:

(2) $$L_{C}=\frac{\mathrm{K\lambda}}{\mathrm{\beta cos\theta}}$$

where *L_C_* is the crystallite size along the c-axis, *K* is the shape factor, *β* is the full width at half maximum (FWHM) of the (002) diffraction peak in radians, and *λ* and *θ* have the same meanings as defined above.

The surface elemental composition and chemical states were investigated by X-ray photoelectron spectroscopy (XPS, K-Alpha, Thermo Scientific, Waltham, MA, USA) equipped with a monochromatic Al Kα X-ray source. Survey spectra and high-resolution spectra were collected at pass energies of 100 and 30 eV, respectively. The binding energy scale was calibrated using the C 1s peak of graphitic carbon at 284.8 eV. High-resolution C 1s spectra were fitted using a Shirley background and Gaussian–Lorentzian peak shapes to analyze the composition and evolution of oxygen-containing functional groups on the carbon fiber surface.

The contact angles of deionized water and diiodomethane on the carbon fiber surface were measured using a contact angle goniometer (DSA25S, KRÜSS, Hamburg, Germany). The surface free energy of the CFs was determined according to the Owens–Wendt model using Equations (3) and (4) [30]:

(3) $${\left(\gamma_{l}^{p}\gamma_{s}^{p}\right)}^{1/2}+{\left(\gamma_{l}^{d}\gamma_{s}^{d}\right)}^{1/2}=\frac{1}{2}\left[\gamma_{l}\left(1+\mathrm{cos\theta}\right)\right]$$

(4) $$\gamma_{s}=\gamma_{s}^{p}+\gamma_{s}^{d}$$

where γ_l_ and γs represent the surface energies of the test liquid and the carbon fiber, respectively; γ_p_ and γ_d_ denote the polar and dispersive components of the surface free energy, respectively; and θ is the measured contact angle.

The interfacial shear strength (IFSS) between the CFs and VER was determined by the microdroplet debonding test [31,32,33] using a composite interface evaluation system (HM410, TOEI KASEI, Nagoya, Japan), as schematically shown in Figure 1b. The resin system was prepared by mixing VER and MEKP at a mass ratio of 100:2 and cured at 140 °C for 2.5 h. At least 10 valid microdroplets were tested for each sample, and the IFSS was calculated according to Equation (5):

(5) $$\mathrm{IFSS}=\frac{F}{\mathrm{\pi dL}}$$

where *F* is the maximum load applied to the resin microdroplet during the debonding process, *d* is the diameter of the single carbon fiber measured by optical microscopy, and L is the embedded length of the cured resin microdroplet on the carbon fiber.

The single-fiber tensile strength was measured using an electronic single-fiber tensile tester (LLY-06E, Laizhou Electronic Instrument Co., Ltd., Laizhou, China) at a crosshead speed of 5 mm/min. As shown in Figure 1c, a single carbon fiber was fixed onto a paper frame using adhesive, and the gauge length was set to 25 mm. Thirty single fibers were tested for each sample, and the average tensile strength was reported.

## 3. Results and Discussion

### 3.1. Optimized Parameters of Electrochemical Treatment

**Figure 2:**
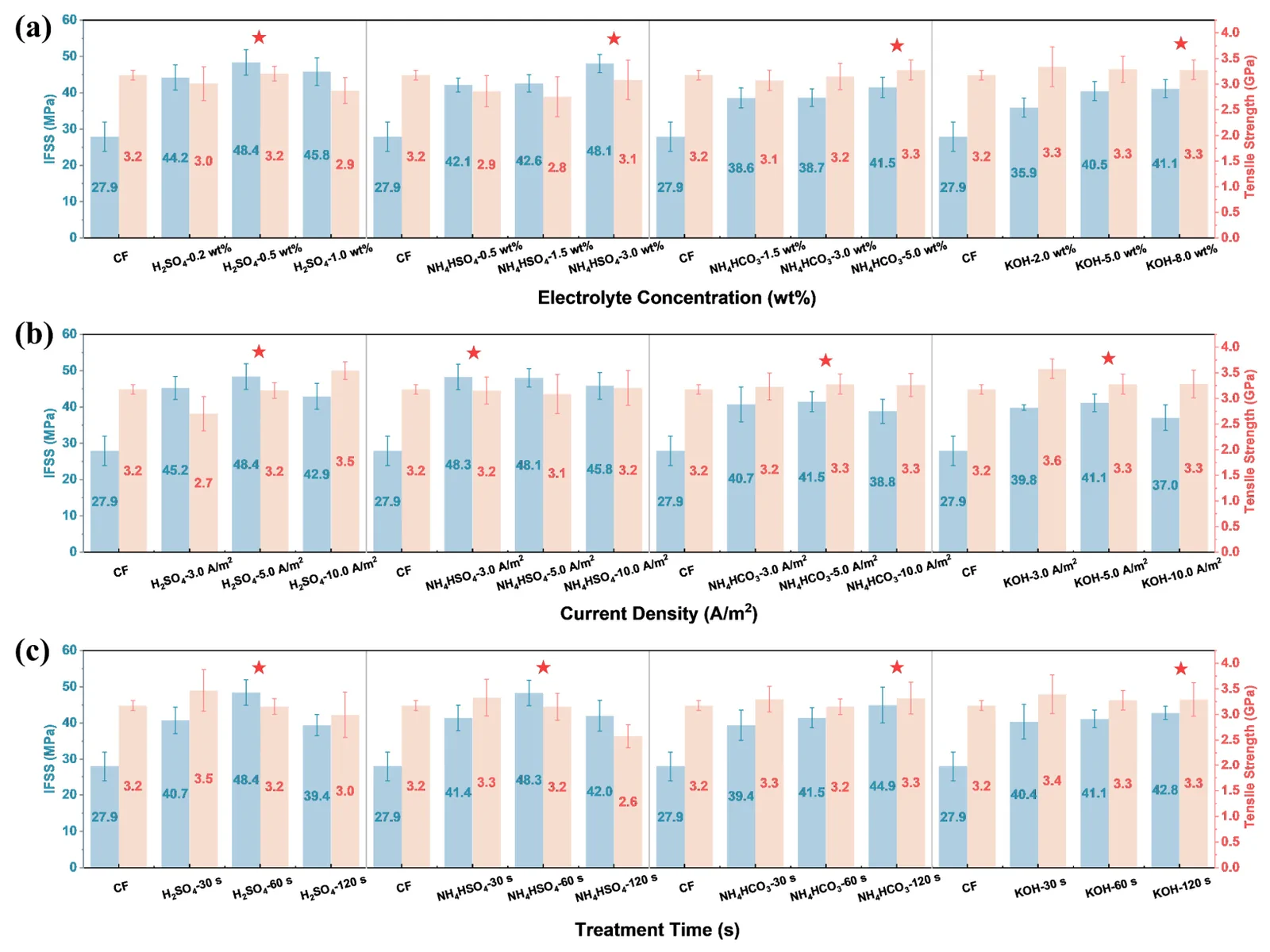
The effects of electrochemical treatment parameters on the IFSS between CFs and the VER matrix and on the single-fiber tensile strength of CFs: electrolyte concentration (**a**), current density (**b**), and treatment time (**c**). Red stars indicate the optimized treatment conditions.

To identify the optimum electrochemical treatment conditions for each electrolyte system, a sequential single-factor optimization was performed by varying electrolyte concentration, current density, and treatment time in sequence. The optimal treatment parameters were determined through a comprehensive evaluation of the trade-off between carbon fiber tensile strength preservation and the enhancement of interfacial shear strength (IFSS) between CFs and the VER matrix, as summarized in Figure 2. As shown in Figure 2a, the optimal electrolyte concentrations for H_2_SO_4_, NH_4_HSO_4_, NH_4_HCO_3_, and KOH were determined to be 0.5, 3.0, 5.0, and 8.0 wt%, respectively. Based on these concentrations, the current density was subsequently optimized, as shown in Figure 2b. A current density of 5 A/m^2^ produced the best overall performance for H_2_SO_4_, NH_4_HCO_3_, and KOH, whereas 3 A/m^2^ was selected for NH_4_HSO_4_. Finally, the treatment time was optimized using the selected electrolyte concentration and current density, as shown in Figure 2c. The optimal treatment times were 60 s for H_2_SO_4_ and NH_4_HSO_4_ and 120 s for NH_4_HCO_3_ and KOH. Under these optimized conditions, the IFSS was significantly enhanced, while the single-fiber tensile strength remained relatively stable. Accordingly, the optimal treatment conditions were determined as follows: 0.5 wt% H_2_SO_4_ at 5 A/m^2^ for 60 s; 3.0 wt% NH_4_HSO_4_ at 3 A/m^2^ for 60 s; 5.0 wt% NH_4_HCO_3_ at 5 A/m^2^ for 120 s; and 8.0 wt% KOH at 5 A/m^2^ for 120 s. All subsequent investigations were based on CFs treated under these optimized conditions. Compared with untreated CF (27.9 MPa), the IFSS values of H_2_SO_4_-CF, NH_4_HSO_4_-CF, NH_4_HCO_3_-CF, and KOH-CF increased to 48.4, 48.3, 44.9, and 42.8 MPa, respectively. The single-fiber tensile strength of untreated CF was 3.2 GPa, while those of the electrochemically treated CFs ranged from 3.1 to 3.3 GPa. The above results indicate that the optimized electrochemical treatments effectively enhanced CF/VER interfacial adhesion without appreciably compromising the intrinsic tensile strength of the CFs.

### 3.2. Surface Morphology and Roughness

**Figure 3:**
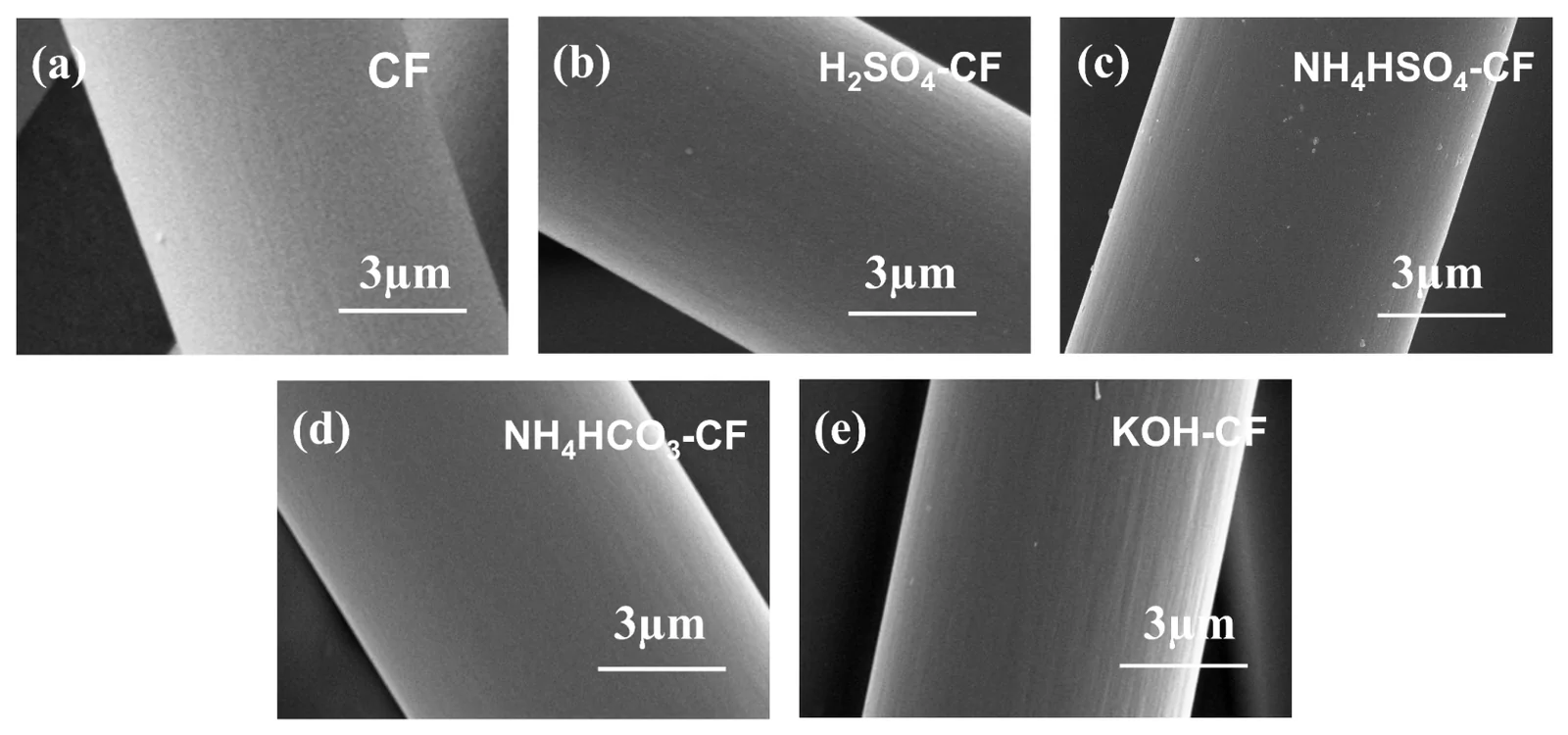
SEM images of untreated CF (**a**), H_2_SO_4_-CF (**b**), NH_4_HSO_4_-CF (**c**), NH_4_HCO_3_-CF (**d**) and KOH-CF (**e**).

**Figure 4:**
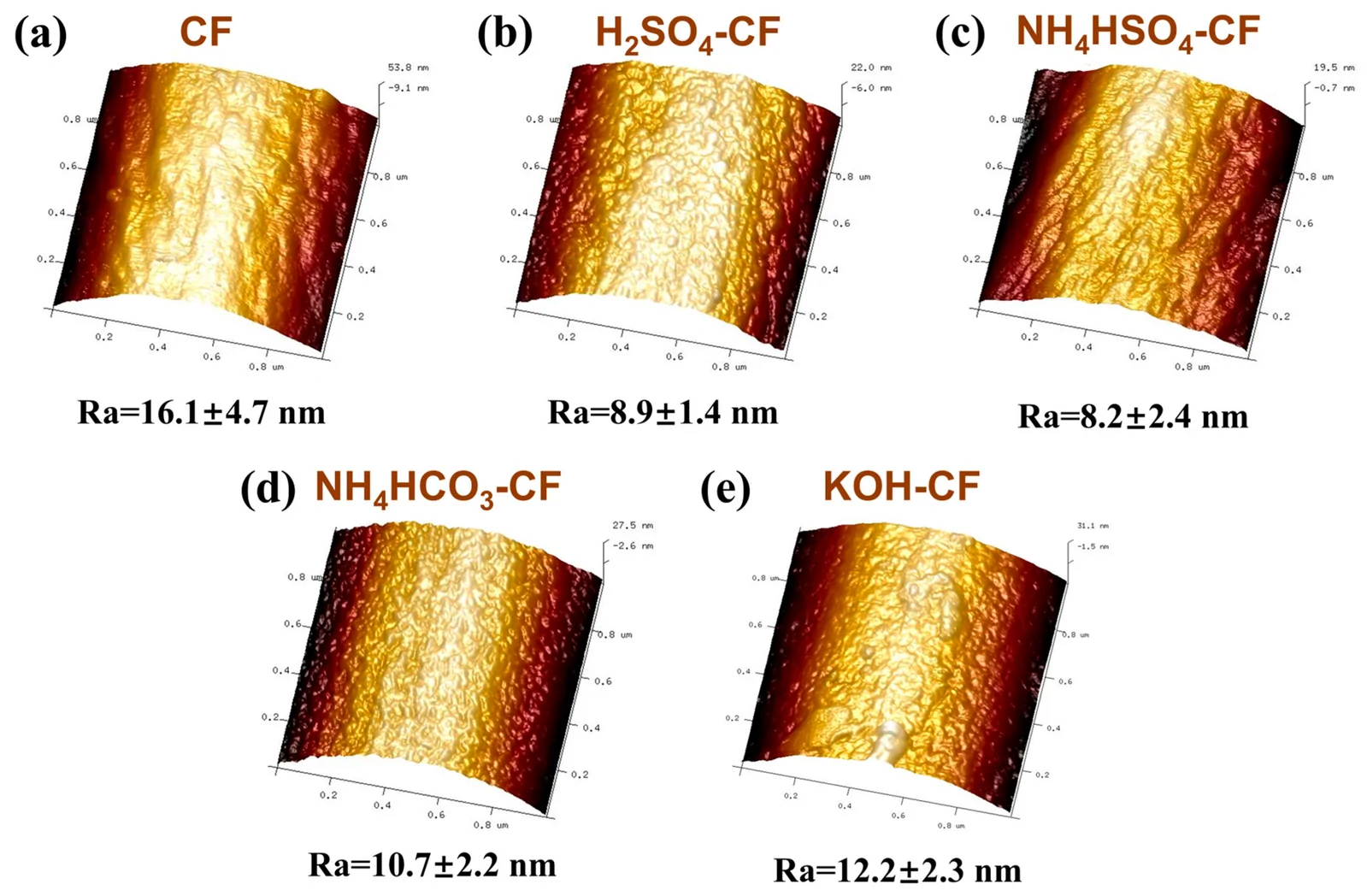
AFM surface topographies and corresponding Ra values of untreated CF (**a**), H_2_SO_4_-CF (**b**), NH_4_HSO_4_-CF (**c**), NH_4_HCO_3_-CF (**d**) and KOH-CF (**e**).

The surface morphology and microscale roughness of untreated and electrochemically treated CFs under the optimized conditions were characterized by SEM and AFM. As shown in Figure 3a, untreated CF exhibits a relatively smooth surface with shallow longitudinal grooves. After electrochemical treatment, no obvious cracks, severe defects, or excessive etching are observed, indicating that the optimized treatment conditions do not cause substantial surface damage. Compared with untreated CF, the treated fibers show clearer axial grooves and more distinct surface textures, suggesting mild surface etching during electrochemical oxidation. AFM analysis further confirms the changes in microscale surface topography. As shown in Figure 4, the Ra value of untreated CF is 11.5 ± 4.7 nm, while those of H_2_SO_4_-CF, NH_4_HSO_4_-CF, NH_4_HCO_3_-CF, and KOH-CF are 8.9 ± 1.4, 8.2 ± 2.4, 10.7 ± 2.2, and 12.2 ± 2.3 nm, respectively. The comparable Ra values and retained axial textures indicate that electrochemical treatment regulates the microscale surface topography rather than causing severe surface roughening or damage. These retained grooves and regulated surface textures may increase the effective contact area between CFs and VER and provide a morphological basis for mechanical interlocking at the interface.

### 3.3. Graphitic Structure and Crystallinity

**Figure 5:**
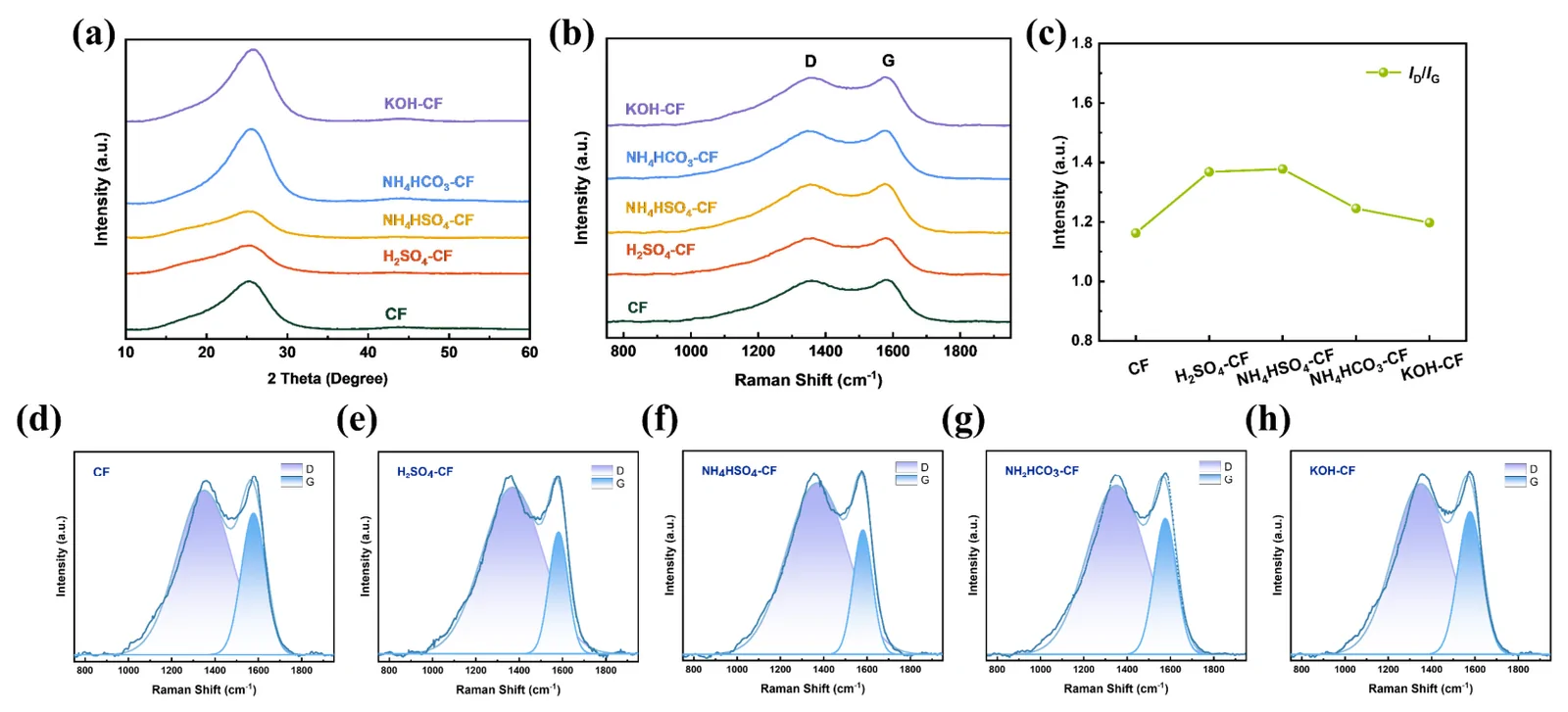
XRD patterns (**a**), Raman spectra (**b**), *I*_D_/*I*_G_ values (**c**) and Raman peak-fitting results (**d**–**h**) of untreated CF, H_2_SO_4_-CF, NH_4_HSO_4_-CF, NH_4_HCO_3_-CF and KOH-CF.

XRD and Raman spectroscopy were used to evaluate the effect of electrochemical treatment on the graphitic structure and crystallinity of CFs. As shown in Figure 5a, all samples exhibit a broad diffraction peak at approximately 25°, corresponding to the graphitic (002) plane of carbon fibers [12,19]. No new diffraction peaks are observed after electrochemical treatment, indicating that the optimized treatment does not significantly alter the bulk crystalline structure of CFs. The structural parameters calculated from the XRD patterns are listed in Table 1. Compared with untreated CF, H_2_SO_4_-CF and NH_4_HSO_4_-CF show slightly increased d_002_ values and decreased Lc values, suggesting that acidic electrolytes induce a certain degree of disturbance in the graphitic stacking structure [19]. In contrast, NH_4_HCO_3_-CF and KOH-CF exhibit relatively lower d_002_ values and higher Lc values, indicating a milder influence on the crystalline structure.

Raman spectra further reveal changes in the surface defect structure of CFs. As shown in Figure 5b, all samples display typical D and G bands, corresponding to disordered carbon structures and sp^2^-hybridized graphitic carbon, respectively [5,19]. The fitted Raman spectra are shown in Figure 5d–h, and the corresponding *I*_D_/*I*_G_ values are summarized in Figure 5c. The *I*_D_/*I*_G_ values of the treated CFs are higher than that of untreated CF, indicating that electrochemical treatment introduces a certain amount of surface defects. Among them, H_2_SO_4_-CF and NH_4_HSO_4_-CF exhibit relatively higher *I*_D_/*I*_G_ values, which is consistent with their increased d_002_ and decreased Lc values. This indicates that the H_2_SO_4_-CF and NH_4_HSO_4_-CF surfaces undergo more extensive oxidation. Their surfaces also contain a relatively higher proportion of non-graphitic structures. These features may contribute to their higher interfacial shear strength with the VER matrix. Overall, the XRD and Raman results demonstrate that electrochemical treatment mainly affects the surface graphitic order while preserving the bulk crystalline structure of CFs, which is consistent with the retained single-fiber tensile strength.

### 3.4. Surface Chemical Composition

**Figure 6:**
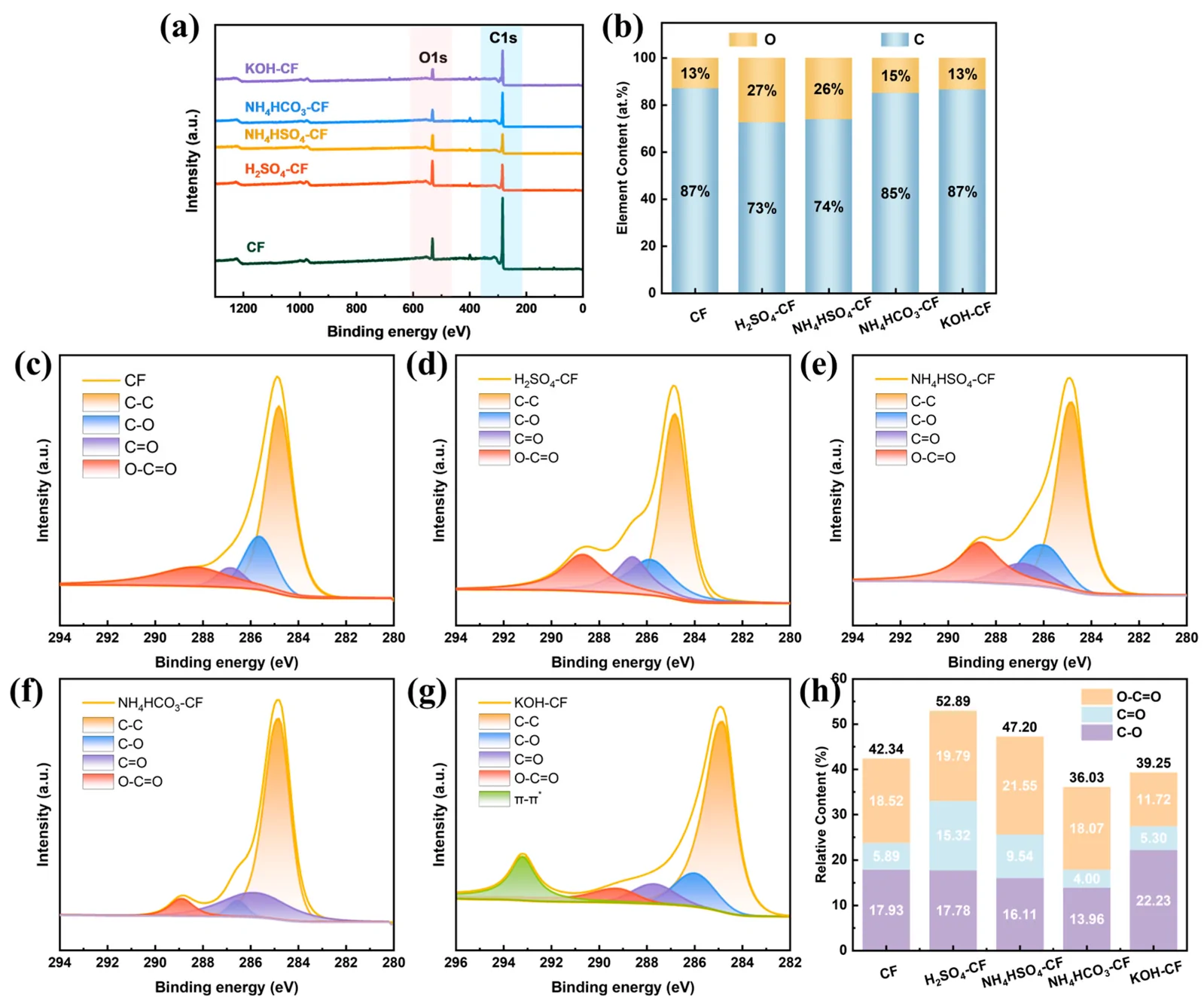
XPS survey spectra (**a**), surface elemental compositions (**b**), deconvoluted high-resolution C 1s spectra (**c**–**g**) and contents of O-containing carbon species (**h**) of untreated CF, H_2_SO_4_-CF, NH_4_HSO_4_-CF, NH_4_HCO_3_-CF and KOH-CF.

To further elucidate the changes in surface chemical composition induced by electrochemical treatment in different electrolyte systems, XPS analysis was performed on untreated and treated CFs. The XPS survey spectra (Figure 6a) show that all samples exhibit predominant C 1s and O 1s peaks, indicating that carbon and oxygen are the major elements present on the fiber surfaces. The XPS survey spectra (Figure 6a) show that all samples exhibit predominant C 1s and O 1s peaks, indicating that carbon and oxygen are the major elements present on the fiber surfaces. As shown in Figure 6b, the surface oxygen content of the untreated CF is 13 at.%, which increases markedly to 27 at.% and 26 at.% after treatment in H_2_SO_4_ and NH_4_HSO_4_ electrolytes, respectively. These results indicate that the acidic electrolytes promote more effective surface oxidation of the CFs. In contrast, the oxygen content increases only slightly to 15 at.% after NH_4_HCO_3_ treatment and remains nearly unchanged at 13 at.% after KOH treatment, suggesting that the alkaline electrolytes produce a relatively mild surface oxidation effect.

The high-resolution C 1s spectra are further deconvoluted to evaluate changes in the surface functional groups, as shown in Figure 6c–g. The fitted C 1s components are assigned primarily to C-C/C=C, C-O, C=O, and O-C=O species [34,35], and the relative contents of the oxygen-containing carbon species, calculated from the fitted peak areas, are summarized in Figure 6h. The surface of the untreated CF is dominated by C-C/C=C bonds and contains relatively low proportions of oxidized carbon species. After electrochemical treatment, the distribution of oxygen-containing functional groups varies markedly with the electrolyte system. The combined relative content of C-O, C=O, and O-C=O species increases from 42.34% for the untreated CF to 52.89% and 47.20% for H_2_SO_4_-CF and NH_4_HSO_4_-CF, respectively, confirming the stronger oxidative functionalization induced by the acidic electrolytes. In particular, the increased proportions of C=O and O-C=O species indicate the formation of more highly oxidized carbon functionalities, which may enhance the surface polarity of the CFs and strengthen their interfacial interactions with the VER matrix. By comparison, NH_4_HCO_3_-CF and KOH-CF exhibit lower combined relative contents of oxygen-containing carbon species, at 36.03% and 39.25%, respectively, indicating a lower degree of oxidative functionalization in the alkaline electrolyte systems. For KOH-CF, although the overall surface oxygen content remains nearly unchanged, the relatively high proportion of C-O species suggests that KOH treatment may preferentially generate or retain less highly oxidized oxygen-containing functionalities rather than forming substantial amounts of C=O and O-C=O species. In addition, KOH-CF exhibits a distinct π-π* shake-up satellite peak in the high-binding-energy region, indicating the presence of aromatic conjugated sp^2^-carbon structures on the fiber surface [29,36]. Overall, these results demonstrate that electrolyte chemistry plays a critical role in regulating both the degree of surface oxidation and the distribution of oxygen-containing functional groups on CF surfaces.

### 3.5. Wettability and Surface Free Energy

**Figure 7:**
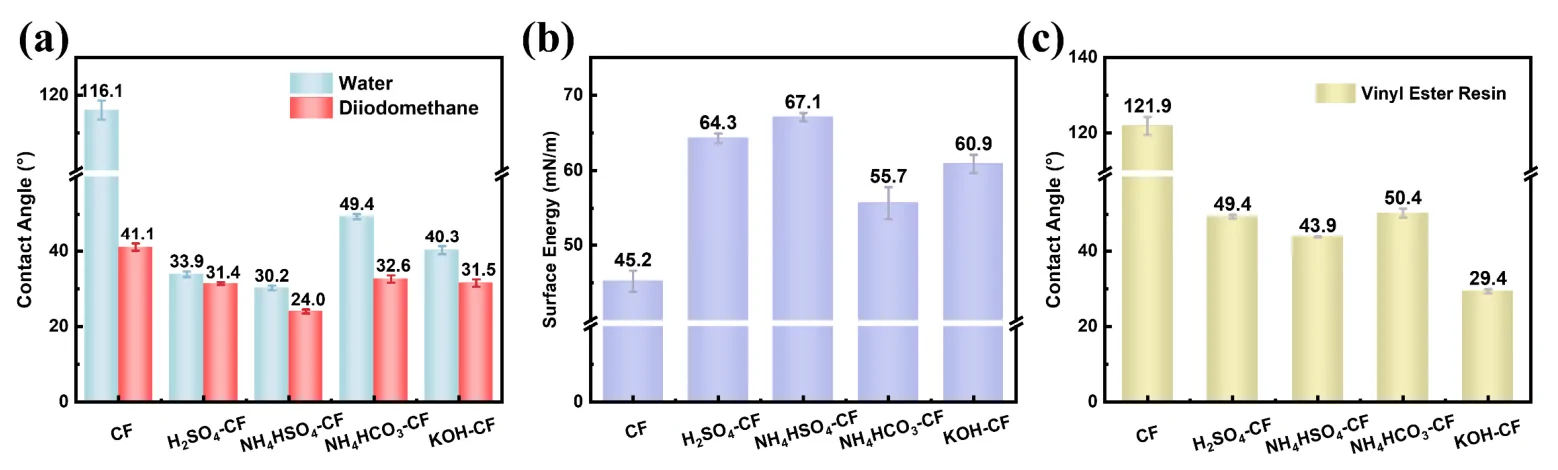
Contact angles of water and diiodomethane (**a**), surface free energy (**b**), and VER contact angle (**c**) of untreated CF, H_2_SO_4_-CF, NH_4_HSO_4_-CF, NH_4_HCO_3_-CF and KOH-CF.

To correlate the surface characteristics with wetting behavior, the contact angles and surface free energies of untreated and electrochemically treated CFs were evaluated, as shown in Figure 7. The untreated CF exhibits a water contact angle of 116.1°, indicating a highly hydrophobic surface. After electrochemical treatment, all treated fibers show markedly lower water contact angles. In particular, the water contact angles of H_2_SO_4_-CF, NH_4_HSO_4_-CF, and KOH-CF decrease to 33.9°, 30.2°, and 40.3°, respectively, indicating substantially enhanced surface hydrophilicity. By comparison, NH_4_HCO_3_-CF exhibits a relatively higher water contact angle of 49.4°, suggesting a less pronounced improvement in hydrophilicity under weakly alkaline conditions. The diiodomethane contact angles also decrease after treatment, further demonstrating the changes in the wetting behavior of the carbon fiber surfaces. Based on the Owens–Wendt model, the surface free energy increases from 45.2 mN/m for the untreated CF to 56.7–67.1 mN/m for the treated fibers, with NH_4_HSO_4_-CF exhibiting the highest value. Overall, the fibers treated in acidic electrolytes exhibit higher surface free energies, consistent with their higher surface oxygen contents determined by XPS. This result also agrees with previous reports showing that oxygen-containing functional groups increase the polar component of surface free energy and thereby improve fiber wettability [27].

To further evaluate the wettability of CFs toward the VER matrix, the contact angle of VER on the fiber surfaces was measured. As shown in Figure 7c, the untreated CF exhibits a high VER contact angle of 121.9°, indicating poor spreading of the resin over the fiber surface. After electrochemical treatment, the VER contact angle decreases substantially to 49.4°, 43.9°, 50.4°, and 29.4° for H_2_SO_4_-CF, NH_4_HSO_4_-CF, NH_4_HCO_3_-CF, and KOH-CF, respectively. Notably, KOH-CF exhibits the lowest VER contact angle, indicating the highest wettability toward the resin matrix. Although KOH-CF shows no obvious increase in surface oxygen content, its markedly improved resin wettability suggests that the wetting behavior of VER is influenced not only by oxygen-containing functional groups but also by the regulated surface topography and fiber-resin interactions. These results demonstrate that electrochemical treatment effectively improves the wettability of CFs toward the VER matrix, thereby providing favorable conditions for enhanced interfacial adhesion.

### 3.6. Interfacial Enhancement Mechanism

**Figure 8:**
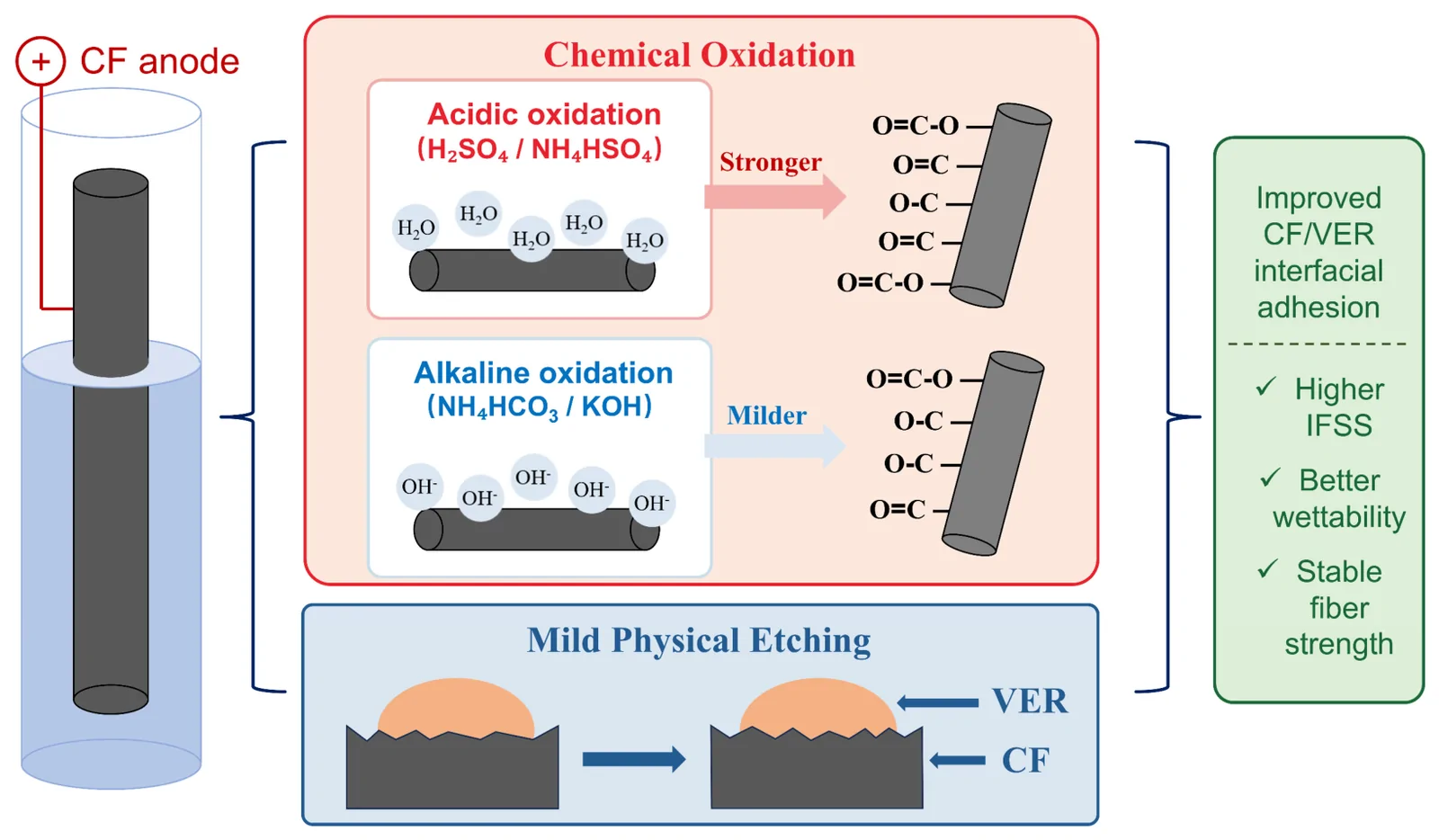
A schematic illustration of the electrochemical surface modification mechanism of CFs involving chemical oxidation and mild physical etching.

The significant improvement in IFSS between electrochemically treated CFs and the VER matrix can be attributed to the synergistic effects of chemical oxidation and mild physical etching [37], as schematically illustrated in Figure 8. During electrochemical treatment, CFs act as the anode, where surface defects, edge carbon atoms, and disordered carbon regions are more susceptible to oxidation [22,28]. In acidic electrolytes, the anodic process can create a stronger oxidative environment at the CF/electrolyte interface, thereby promoting the formation of oxygen-containing functional groups, such as C-O, C=O, and O-C=O [38]. This increases the surface oxygen content and surface free energy of CFs. In contrast, under alkaline conditions, the anodic process is more closely related to OH^−^-involved oxidation reactions, resulting in a milder oxidative modification effect and a smaller increase in surface oxygen content. In addition to chemical oxidation, electrochemical treatment induces mild physical etching and regulates the surface textures of CFs. The retained axial grooves and modified microscale topography can facilitate VER spreading and local infiltration, thereby enhancing mechanical interlocking. Therefore, chemical oxidation enhances surface polarity and interfacial interactions, whereas mild physical etching and surface texture regulation promote VER spreading and mechanical interlocking; together, these effects improve CF/VER interfacial adhesion while preserving the intrinsic fiber strength under optimized conditions.

## 4. Conclusions

This study systematically compares the effects of H_2_SO_4_, NH_4_HSO_4_, NH_4_HCO_3_, and KOH electrolytes on the electrochemical surface modification of CFs and their interfacial adhesion with the VER matrix. Under the optimized treatment conditions, the fiber morphology remains well preserved without obvious cracks, severe defects, or excessive etching. SEM and AFM results indicate that electrochemical treatment regulates the microscale surface topography while retaining axial surface textures, rather than causing severe surface roughening or damage. Raman and XRD analyses further confirm that the bulk graphitic/crystalline structure of CFs is largely preserved, which is consistent with the retained single-fiber tensile strength of 3.1–3.3 GPa. Acidic electrolytes induce stronger oxidative functionalization, increasing the surface oxygen content from 13 at.% to 27 at.% and 26 at.% for H_2_SO_4_-CF and NH_4_HSO_4_-CF, respectively, whereas NH_4_HCO_3_ and KOH produce milder oxidation. Nevertheless, all treatments substantially improve fiber wettability, as evidenced by the decrease in water contact angle from 116.1° to 30.2–49.4°, the increase in surface free energy from 45.2 to 56.7–67.1 mN/m, and the decrease in VER contact angle from 121.9° to 29.4–50.4°. Consequently, the IFSS increases from 27.9 MPa for untreated CF to 48.4, 48.3, 44.9, and 42.8 MPa for H_2_SO_4_-CF, NH_4_HSO_4_-CF, NH_4_HCO_3_-CF, and KOH-CF, respectively. The interfacial enhancement is attributed to the synergistic effects of chemical oxidation and mild physical etching. Chemical oxidation increases surface polarity and interfacial interactions, while mild physical etching and regulated surface textures improve resin spreading and mechanical interlocking. Acidic electrolytes are more effective in promoting oxidative functionalization, whereas alkaline electrolytes can still achieve substantial interfacial improvement through mild etching and enhanced resin wettability. In summary, the combined characterization results obtained in this work provide useful guidance for optimizing electrolyte systems and treatment parameters to achieve enhanced CF/VER interfacial adhesion and facilitate the development of high-performance CFRVEs.

## Figures and Tables

**Table 1:** Interlayer spacing (d_002_) and crystallite size (Lc) of untreated and electrochemically treated CFs calculated from XRD patterns.

Samples	d_002_ (nm)	L_C_ (nm)
Untreated CF	0.363	1.038
H_2_SO_4_-CF	0.374	0.817
NH_4_HSO_4_-CF	0.371	0.855
NH_4_HCO_3_-CF	0.355	1.193
KOH-CF	0.354	1.122

## Data Availability

The original contributions presented in this study are included in the article. Further inquiries can be directed to the corresponding author.

## References

[1] Chae H., Newcomb B., Gulgunje P., Liu Y., Gupta K., Kamath M., Lyons K., Ghoshal S., Pramanik C., Giannuzzi L. (2015). High strength and high modulus carbon fibers. Carbon.

[2] Hiremath N., Young S., Ghossein H., Penumadu D., Vaidya U., Theodore M. (2020). Low cost textile-grade carbon-fiber epoxy composites for automotive and wind energy applications. Compos. Part B Eng..

[3] do Amaral Junior M.A., Matsushima J.T., Rezende M.C., Gonçalves E.S., Marcuzzo J.S., Baldan M.R. (2017). Production and Characterization of Activated Carbon Fiber from Textile PAN Fiber. J. Aerosp. Technol. Manag..

[4] Mamat R., Rashid M.I.M., Syahir A.Z., Erdiwansyah, Yusop A.F., Tamimi A. (2025). Carbon fibre for applications in aerospace: A review. J. Alloys Metall. Syst..

[5] Woodgate C., Trask R., Shaffer M., Eichhorn S. (2024). Raman spectroscopic stress mapping of single high modulus carbon fibre composite fragmentation in compression. Compos. Sci. Technol..

[6] Zhang J., Lin G., Vaidya U., Wang H. (2023). Past, present and future prospective of global carbon fibre composite developments and applications. Compos. Part B Eng..

[7] Alia C., Jofre-Reche J.A., Suárez J.C., Arenas J.M., Martín-Martínez J.M. (2018). Characterization of the chemical structure of vinyl ester resin in a climate chamber under different conditions of degradation. Polym. Degrad. Stab..

[8] Kandelbauer A., Tondi G., Zaske O.C., Goodman S.H., Dodiuk H., Goodman S.H. (2014). Unsaturated Polyesters and Vinyl Esters. Handbook of Thermoset Plastics.

[9] Li H. (1998). Synthesis, Characterization and Properties of Vinyl Ester Matrix Resins. Doctoral Dissertation.

[10] Ran X., Qin J., Wang M., Jiang Q., Zeng Y., Liu W. (2024). Synergistic toughening of carbon fiber/vinyl ester resin composites with core–shell rubber particles and polyphenylene sulfide veils. Polym. Compos..

[11] Zou W., Tong Y., Wang Y., Xu L. (2025). Antagonistic effect of intermediate modulus carbon fibre structures on axial compressive failure of composites. Compos. Part A Appl. Sci. Manuf..

[12] Sharma M., Gao S., Mäder E., Sharma H., Wei L.Y., Bijwe J. (2014). Carbon fiber surfaces and composite interphases. Compos. Sci. Technol..

[13] Peng X., Wu Y., Wei Z. (2024). Research progress on the surface modification of carbon fiber. RSC Adv..

[14] Hu M., Leng C., Zhang B., Ma X., Xu M., Guo N., Ai L., Ma Q., Yuan D., Zhang S. (2025). Electrochemical graft of polymer molecules on carbon fibers surfaces to construct high-performance carbon fiber/epoxy composites with micrometer-scale interphases. Compos. Part B Eng..

[15] Lee Y., You J., Kim M., Kim T., Lee S., Bang J., Park J. (2019). Highly improved interfacial affinity in carbon fiber-reinforced polymer composites via oxygen and nitrogen plasma-assisted mechanochemistry. Compos. Part B Eng..

[16] Zhang Z., Wilson J.L., Kitt B.R., Flaherty D.W. (2021). Effects of Oxygen Plasma Treatments on Surface Functional Groups and Shear Strength of Carbon Fiber Composites. ACS Appl. Polym. Mater..

[17] Srivastava A.K., Desai U., Singh A. (2023). Effect of graphene coating on modified and pristine carbon fibers on the tribological response of carbon fiber epoxy composites. Compos. Part B Eng..

[18] Wang S., Zhang S., Yang Y., Dong Z., Wang G. (2022). Direct electrochemical grafting of crystalline PAEK macromolecule on carbon fiber to enhance the interfacial properties of PEEK/CF composites. Compos. Sci. Technol..

[19] Chen L., Ma L., Xiao S., Li L., Shen Z., Wang Y., Chen M. (2025). Subsurface microstructural differences and mechanical behaviors of PAN-based graphitic carbon fibers during electrochemical treatment. Carbon.

[20] Sun Y., Lu Y., Yang C. (2019). Stripping mechanism of PAN-based carbon fiber during anodic oxidation in NaOH electrolyte. Appl. Surf. Sci..

[21] Tran N.T., Patterson B.A., Kolodziejczyk A.G., Wu V.M., Knorr D.B. (2019). Electrochemical Surface Treatment of Discontinuous Carbon Fibers. Langmuir.

[22] Shi P., Zhu Y., Yan C., Liu D., Xu H. (2025). Electrochemically oxidized carbon fiber surfaces: Mechanism-driven models for enhanced surface engineering. Mater. Today Chem..

[23] Ma C.-p., Zhang Y., Zhang H., Gao A.-j., Wang Y. (2023). Effects of Acid–Base Properties of Ammonium Salt Electrolyte on Electrochemical Surface Activation of Carbon Fibers. Fibers Polym..

[24] Bauer M., Beratz S., Ruhland K., Horn S., Moosburger-Will J. (2020). Anodic oxidation of carbon fibers in alkaline and acidic electrolyte: Quantification of surface functional groups by gas-phase derivatization. Appl. Surf. Sci..

[25] Huang C., Zhang P., Liu Z., Sun M., Liu H., Sun J., Li B., Zhao Y., Bao J. (2024). Effect of electrochemical anodic oxidation modification on the interfacial properties of carbon fiber reinforced polyimide composites. Polym. Compos..

[26] Qiu S., Wang J., Zhang D., Van Vuure A.W., Seveno D., Fuentes C.A. (2019). Wetting dynamics and surface energy components of single carbon fibers. J. Colloid. Interface Sci..

[27] Xu Z., Chen L., Huang Y., Li J., Wu X., Li X., Jiao Y. (2008). Wettability of carbon fibers modified by acrylic acid and interface properties of carbon fiber/epoxy. Eur. Polym. J..

[28] Xiao J., Tian S., Zhou H., Gao A., Xu L. (2023). Construction of Polar Functional Groups on the Surface of a High-Modulus Carbon Fiber and Its Effect on the Interfacial Properties of Composites. ACS Omega.

[29] Cao Q., Lucktong J., Shao Z., Chang Y., Venton B.J. (2021). Electrochemical treatment in KOH renews and activates carbon fiber microelectrode surfaces. Anal. Bioanal. Chem..

[30] Kaelble D.H. (2008). Dispersion-Polar Surface Tension Properties of Organic Solids. J. Adhes..

[31] Li H., Liu C., Zhu J., Sun J., Huan X., Geng H., Li T., Ge L., Jia X., Yang X. (2024). Spider silk-inspired heterogeneous interphase featuring hybrid interaction for simultaneously improving the interfacial strength and fracture toughness between carbon fiber and epoxy by regulating hydrogen bond density. Compos. Part B Eng..

[32] Zhi C., Long H., Miao M. (2017). Influence of microbond test parameters on interfacial shear strength of fiber reinforced polymer-matrix composites. Compos. Part A Appl. Sci. Manuf..

[33] Kang S., Lee D., Choi N. (2009). Fiber/epoxy interfacial shear strength measured by the microdroplet test. Compos. Sci. Technol..

[34] Yue Z.R., Jiang W., Wang L., Gardner S.D., Pittman C.U. (1999). Surface characterization of electrochemically oxidized carbon fibers. Carbon.

[35] Morgan D.J. (2021). Comments on the XPS Analysis of Carbon Materials. C.

[36] Hanser S.M., Shao Z., Zhao H., Venton B.J. (2024). Electrochemical treatment in KOH improves carbon nanomaterial performance to multiple neurochemicals. Analyst.

[37] Kim D.-K., An K.-H., Bang Y.H., Kwac L.-K., Oh S.-Y., Kim B.-J. (2016). Effects of electrochemical oxidation of carbon fibers on interfacial shear strength using a micro-bond method. Carbon. Lett..

[38] Kainourgios P., Kartsonakis I.A., Dragatogiannis D.A., Koumoulos E.P., Goulis P., Charitidis C.A. (2017). Electrochemical surface functionalization of carbon fibers for chemical affinity improvement with epoxy resins. Appl. Surf. Sci..

